# Grisel's syndrome: a rare complication following traditional uvulectomy

**DOI:** 10.11604/pamj.2015.20.62.5930

**Published:** 2015-01-22

**Authors:** Abdelhakim Elyajouri, Abdellah Assermouh, Rachid Abilkacem, Aomar Agadr, Chafiq Mahraoui

**Affiliations:** 1 Pediatric Unit, Mohammed V Military Hospital, Faculty of Medicine, Mohammed V University, Rabat, Morocco; 2 Pneumo-allergology unit of Rabat children hospital, Faculty of Medicine, Mohammed V University Rabat, Morocco

**Keywords:** Grisel′s syndrome, uvulectomy, torticollis

## Abstract

A case is reported of an eight-month-old female patient who had traditional uvulectomy for sore throat complicated by Grisel's syndrome. She was admitted into the hospital one week after uvulectomy with Torticolis. Grisel's syndrome is a nontraumatic atlantoaxial subluxation, usually secondary of an infection or an inflammation at the head and neck area, or after surgery in the same area. Patients typically suffer from painful torticollis. Diagnosis of Grisel's syndrome is largely based on suspicion of the patient who has recently undergone surgery or has a history of an infection in head and neck area. Physical examination and imaging techniques assist in diagnosis. Thus, clinicians should be aware of acute nontraumatic torticollis if patient had a recent surgery in the head or neck area or undergone an upper respiratory tract infection. In this paper, a case of an eight-month-old female patient who had Grisel's syndrome after uvulectomy is discussed. This case is reported to highlight this neurogical threatening complication following traditional uvulectomy as well as highlighting the unnecessary morbidity and mortality associated with this persisting mode of treatment in Africa.

## Introduction

Traditional African practitioners continue to perform uvulectomies at the request of their patients despite severe complications noted by physicians [1]. These severe complications may require hospitalization[2]. Traditional uvulectomy is an African surgical practice that has been well documented in Kenya, Sierra Leone, Tanzania, Ethiopia, South Africa and Nigeria [2–8]. It has also been reported in Israel, Maghreb region [9] and Saudi Arabia [10]. It is an unnecessary and potentially dangerous practice. Grisel's syndrome known as the subluxation of atlas and axis was firstly described by Sir Charles Bell in 1830 in a syphilitic patient with pharyngeal ulceration. Bell reported death in his patient by spinal cord compression due to atlanto-axial subluxation and autopsy report showed the erosion of the axis transverse ligament [11, 12]. In 1930, the French physician Grisel who gave his name to the syndrome reported 2 cases having this syndrome which had developed after nasopharyngeal inflammation [13, 14]. Increased flexibility of the atlantoaxial joint ligaments has been implicated as the underlying reason for this syndrome [11–15]. Although the etiopathogenesis of this syndrome has not been exactly proven, risk factors reported are: pediatric age group, history of pharyngitis, adenotonsillitis, abscess of peritonsillar and cervical region, otitis media, trauma, any upper respiratory tract infection, genetic disorders, and to-be-performed head and neck surgery. There is not a definite consensus criterion for the diagnosis and treatment, but early diagnosis is very important for prognosis. Late diagnosis and inadequate treatment may cause neurological sequel and/or painful and lasting deformity of the neck [13, 15]. Treatment of the acute form is easy, consisting of bed rest, antibiotics, anti-inflammatory agents, immobilization, and/or simple traction. To our knowledge, this represents a rare case report of such complication resulting from uvulectomy.

## Patient and observation

**Figure 1:**
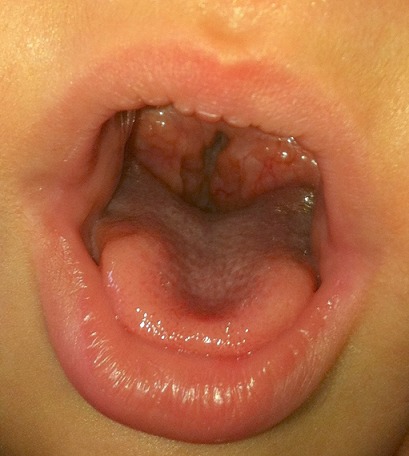
Photo of the patient's throat following traditional uvulectomy

**Figure 2:**
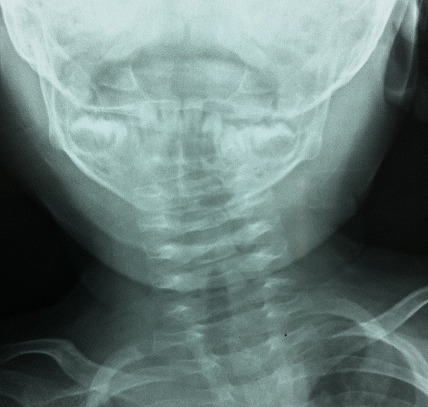
Cock-robin position on anterior-posterior direct cervicography

**Figure 3:**
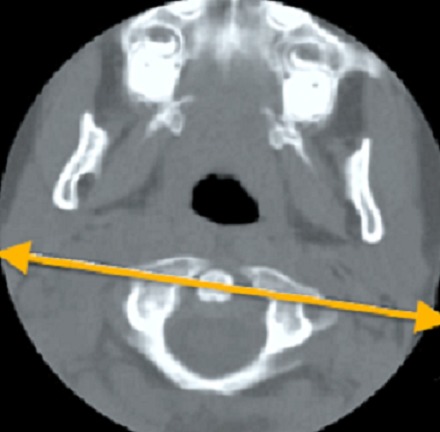
Rotator atlantoaxial subluxation

An 8-month-old female patient who had traditional uvulectomy for loss of appetite was admitted with painful torticollis on the postoperative one week. Oropharyngeal examination revealed an inflammed oropharynx, and **inflamed** uvula stump (Figure 1). The neurological examination was normal. In the neck there were multiple tender small (< 2 cm). Both Direct cervicography (DCG) and cervical computerized tomography (CT) were performed for diagnosis. Cock-robin position was detected on anterior-posterior DCG (Figure 2). Rotator atlantoaxial subluxation through the asymmetric thickening of soft tissue was observed on cervical CT (Figure 3). Antibiotic (amoxicillin-clavulanic acid) and anti-inflammatory (ibuprofen) treatment were given to the patient, and absolute bed rest and immobilization with a cervical collar was recommended. The symptoms gradually decreased by treatment and it took 5 days to full recovery. Antibiotic therapy was continued to the 10th day while the anti-inflammatory therapy continued until to 7^th^ day.

## Discussion

Uvulectomy in Africa is performed as a ritual custom on both boys and girls during the first or second years of life [9]. In some parts of Africa among the Hausa ethnic group, it is systematically performed on the seventh day after birth, during naming ceremony to prevent death due to a “swelling of the uvula” [2, 16]. Another reason for this practice is a curative one, both for children and adults who complain of sore throat, vomiting, diarrhea, anorexia, rejection of breast-feeding by a child, delayed growth and fever. Occasionally it is done to prevent throat infections and other disorders associated with the throat because it is believed that elongated uvula is the root cause of all throat problems [6, 8]. Uvulectomy is an unnecessary and potentially dangerous mutilation as it results in various complications including haemorrhage, septicemia, cellulitis of the neck, peritonsillar abscess, pneumothorax, parapharyngeal abscess and pharyngo-laryngocele [7]. In the reported case the uvulectomy done by the traditional practitioner was complicated by Grisel's syndrome. Grisel's syndrome is atlantoaxial joint subluxation without trauma or concomitant bone pathology generally occurs in childhood and etiopathogenesis has not been clearly explained. The atlas is a ring of bone with flat, slanting articulating facets on the lateral masses. The articulating facets form synovial joints with the corresponding anterior articulating surfaces of the axis; each has a loose capsule allowing maximum rotation with minimal lateral displacement. The primary stabilizer of the atlanto-axial joint is the transverse ligament; it is attached to the lateral posterior portion of the anterior arch of the atlas and forms the posterior support of the odontoid process, preventing excessive shift of C-1 on C-2. The paired alar ligaments are secondary stabilizers preventing excessive rotation. Numerous venous tributaries drain the posterior superior region of the nasopharynx. Pharyngovertebral veins cross the prevertebral fascia and drain into the peri-odontoidal plexuses, ultimately emptying into the upper cervical epidural venous sinuses. A possible pathway for the spread of inflammation to the atlanto-axial ligaments has been demonstrated via direct anastomoses between lymphatic vessels and pharyngo-vertebral veins. Naso-pharyngeal inflammation causes hyperaemia that may weaken the transverse and alar ligaments and the articular capsules resulting in atlanto-axial instability, followed by pathological rotation of the atlas on the axis [17]. This condition begins as a typical torcicollis caused by spasms in irritated neck muscles, resulting, if prolonged, in distension of the ligaments. This second hypothesis, which presumes lax ligaments as a prerequisite for Grisel's syndrome, is predominantly seen in children [18]. Moreover, there is an increased risk of non-traumatic atlanto-axial subluxation in Down's syndrome, a condition in which lax ligaments are a recognized feature [19].

Typical patients show neck stiffness and torticollis associated painful neck movements. This syndrome can be seen after rhinopharyngitis, cervical osteomyelitis, rheumatic conditions, and surgical procedures such as adenoidectomy, tonsillectomy, repairment of choanal atresia, and mastoidectomy. Also, hard object shock, mumps, or retropharyngeal abscess have been reported [11, 13–15]. It can be seen with congenital syndromes such as Down and Marfan which have increased ligament looseness [11, 12]. Another factor of interest is increased atlantoaxial distance in Down's syndrome [14]. The other risk patient groups are characterized by spinal instability such as Morquio's syndrome, Klippel-Feil syndrome, osteogenesis imperfecta, and neurofibromatosis. Other pathologies with similar symptoms must be ruled out as like the cervical bone anomalies, posterior fossa tumors, or cervical trauma [12]. In our opinion, the provoking factors were surgery (uvulectomy) and inflammation in our case. Torticollis can spontaneously occur after pharyngitis and minor trauma to the neck; the differential diagnosis of torticollis requires a complete medical history, clinical and radiological examination. It should be kept in mind that the neck pain, limited neck motion, cock-robin position, and torticollis can be early signs of Grisel's syndrome after uvulectomy. Sensitive palpable c2 spinous process is a strong indicator of atlantoaxial subluxation (Sudeck's sign) [14, 20, 21]. Patients may feel neck pain and tingling at the upper and lower extremities in the neck flexion (Lhermitte's sign) [21]. Although less than 15% of patients show neurological signs and symptoms, extreme consequences such as quadriplegia and sudden death [21]. Radiological evaluation is very important for early diagnosis because increased atlantoodontoid distance could be detected in the lateral projection. Normal atlantoodontoid distance is ≤3 mm in adults and ≤5 mm in children [22]. CT and magnetic resonance imaging (MRI) are excellent diagnostic tools to show the deep neck infection and relationship between ligamentous and bone structure of the spine [15]. Dynamic CT can cause neurological complications [15]. Inflammatory indicators are not specific for this syndrome [14].

If effective and immediate treatment does not commence, subluxation can develop [12]. Consultation of the patient with relevant branches (physical medicine and rehabilitation, neurosurgery and pediatrics, etc.) and evaluation is very important for in-time treatment and preventing significant neurological deficits [14]. Treatment must be quickly initiated to avoid neurological complications [15]. Conservative treatment including bed rest, antimicrobial therapy, muscle relaxants, anti-inflammatory agents, external fixation, cervical traction, and soft or hard collar is of great importance at this early period that has rotational deformity without subluxation [14, 22]. The subluxation can develop after painful torticollis and/or fever in patients. If muscle spasm lasts longer than 24 hours, adding diazepam to early treatment may prevent the poor prognosis. In conclusion, diagnosis of the Grisel's syndrome is largely based on suspicion of the patient who has recently underwent surgery or history of infection in head and neck region [14, 15]. Early diagnosis of the atlantoaxial subluxation is required for careful clinical and radiological evaluation and consultation with relevant branches. Early intervention is critical for prognosis; conversely, delay in diagnosis can be dramatic. Therefore, clinicians should be aware of acute nontraumatic torticollis after recently applying the head and neck surgery [15].

## Conclusion

This case is reported to highlight this neurogical threatening complication following traditional uvulectomy. It should be discouraged and laws promulgated to stop it and programs for training and retraining of the practitioners instituted to improve their practice and safeguard people in our society.
